# Rapid carbon accumulation following managed realignment on the Bay of Fundy

**DOI:** 10.1371/journal.pone.0193930

**Published:** 2018-03-21

**Authors:** Jan T. Wollenberg, Jeff Ollerhead, Gail L. Chmura

**Affiliations:** 1 Department of Geography, McGill University, Montreal, Québec, Canada; 2 Department of Geography and Environment, Mount Allison University, Sackville, New Brunswick, Canada; Centro de Investigacion Cientifica y de Educacion Superior de Ensenada Division de Fisica Aplicada, MEXICO

## Abstract

Salt marshes are highly effective carbon (C) sinks and have higher rates of soil C burial (per square meter) than terrestrial ecosystems. Marsh reclamation and anthropogenic impacts, however, have resulted in extensive losses of salt marshes. Restoration of marshes drained and “reclaimed” for agriculture (referred to in Canada as dykelands) and degraded marshes can generate C credits, but only if C burial is reliably quantified. To date, studies reporting on C burial rates have been limited primarily to restored marshes which are more than 10 years old. Here we report on a study which assessed C burial six years after the return of tidal flooding to a section of dykeland in Aulac, New Brunswick on Canada’s Bay of Fundy. The C burial rate in the restored marsh averaged 1 329 g C m^-2^ yr^-1^, more than five times the rate reported for a nearby mature marsh. Carbon density in the recovering marsh was relatively consistent with depth and although salt marsh cordgrass (*Spartina alterniflora*) became established in 2012, the bulk of the C in the new marsh deposit is assumed to be allochthonous. Financial constraints are a barrier to marsh restoration projects and C markets could provide a considerable source of funding for restoration work in the future. For marsh restoration projects to be recognized in C crediting systems, however, it must also be demonstrated that the allochthonous C would not otherwise have been sequestered; the potential for this is discussed.

## Introduction

Salt marshes are well recognized for their value as highly effective carbon (C) sinks [1, 2]. The C stored in the biomass and sediment of vegetated coastal ecosystems is collectively described as “blue carbon” [2, 3]. Carbon dioxide (CO_2_) is fixed by marsh vegetation through photosynthesis and stored in both aboveground and belowground biomass, thus contributing to autochthonous C storage (i.e., C sources from within the marsh). In contrast, allochthonous C sources are available through regular tidal flooding which delivers sediment and organic C from outside the ecosystem boundaries to the marsh [2, 4]. Marsh vegetation contributes indirectly to storage of allochthonous C by trapping suspended sediment. The persistence of anaerobic conditions results in low rates of decomposition [5, 6] so that salt marshes accumulate organic matter which, along with mineral sediment import, allows marshes to accrete vertically and laterally [7, 8]. Provided the rate of sea-level rise is not too great, marshes can thus continue to accrete in step with the pace of sea level rise [9, 10].

These factors mean that on an areal basis, salt marsh soils accumulate organic C at a faster rate than soils of terrestrial ecosystems [2]. Average (±se) carbon burial rates of 5.1±1.0, 4.0±0.5, and 4.6±2.1 g m^-2^ yr^-1^ are characteristic of temperate, tropical and boreal forests, respectively [11, 12], although temporarily higher rates of 55 g C m^-2^ yr^-1^ have been reported in abandoned agricultural soils that were allowed to return to native temperate forest vegetation [11]. In contrast, salt marsh soils store organic C at a rate of 218 ±24 g m^-2^ yr^-1^, with a range of 19–1713 g C m^-2^ yr^-1^ [2].

Salt marsh reclamation, however, has resulted in the extensive loss of this important C sink for centuries [13, 14, 15]. In Britain, dyking has occurred since Roman times [16]. In France and the Netherlands, dykes were built for agricultural conversion and flood defence as early as the 11^th^ century [14] and these practices have been used for 400 years in Eastern North America [13, 14, 17]. In Canada, it is estimated that 77% (330 km^2^) of pre-existing marsh on the Bay of Fundy has been drained [18] while an estimated 25–50% of global salt marsh area has been lost over the past century [15]. Thus, restoring salt marsh habitat can help to reduce atmospheric concentrations of CO_2_.

With increasing rates of sea level rise and the possibility of increased storm severity due to climate change [19, 20], policy makers are beginning to reconsider the long-term cost effectiveness associated with maintaining dykes, sea walls, and “hard” defences [21, 22]. In response to rising costs, coastal managers are turning to a process known as managed realignment (MR) which involves the breaching, removal, or landward movement of a sea defence structure to restore tidal influence and promote the restoration of intertidal habitat [21]. Managed realignment projects have demonstrated that limited management or pre-treatment is required and that when tidal flow can return to a drained, low-lying agricultural field, new salt marsh can become established within less than 10 years [23, 24, 25, 26]. The success of MR projects has been demonstrated by comparing vegetation [27, 28, 29] and hydrology [25] of the recovering site to a reference site. Around Essex in the UK, restored marshes which were dyked and drained since medieval times are now 82% to 96% similar to reference sites [27].

Coastal managers in Europe are increasingly examining the potential reversal of previous marsh reclamation [30], but marsh restoration has received relatively little interest to date in Canada. Yet, Bay of Fundy dykelands are well suited for MR and restoration projects. Byers and Chmura [28] studied the distribution of vegetation with respect to elevation in two recovered marshes on the Bay of Fundy following the unintentional breaching of dykes. The large elevation range observed for the native *Spartina alterniflora* [28], coupled with high rates of sediment deposition observed in Fundy marshes [31], indicate that marsh recovery is likely to be successful without the addition of any fill material prior to restoration [28]. Although rates of C sequestration in Eastern Canada have been assessed in mature salt marshes e.g. [1, 31], there has been little study of C accumulation rates in recovering marshes, particularly in the short-term (<10 years) following restoration.

In 2010, Ducks Unlimited Canada (DUC) performed a controlled breach of two sections of dyked land on the Bay of Fundy near Aulac, New Brunswick [32]. A new dyke was constructed inland to protect remaining pastured dykeland while allowing tidal flooding to return to the area between the old and new dykes. Here we calculate rates of C burial in the recovering marsh.

## Methods

### Study site

The study site is a section of Aulac dykelands (45°51’15.38"N, 64°17’47.59"W) located in the upper Bay of Fundy where the tidal amplitude is ~12 m [33]. The surrounding region is dominated by dykeland agriculture, primarily for hay production and pasture but with some growing of corn, cereal crops, and sod, while the upland regions are dominated by northern tree species such as spruce, fir, birch, and poplar. The towns of Sackville, NB (population ~5,300) and Amherst, NS (population ~9,400) flank the dykelands and there are other low-density residential developments around the upper Bay (primarily detached homes in the surrounding rural areas).

**Fig 1:**
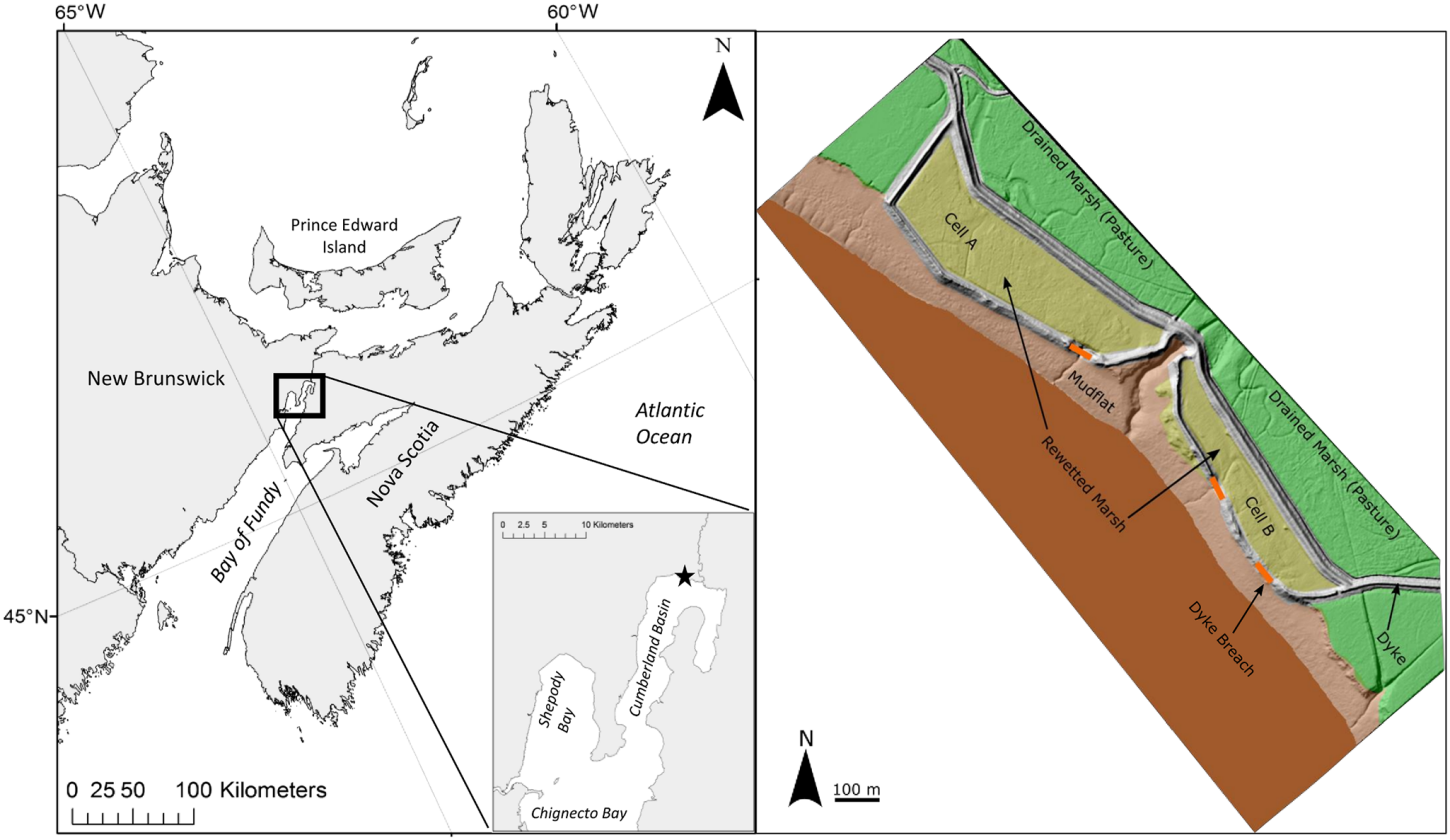
Location of restored marsh, its cells and breach points, Aulac, NB. Left, location of site on coast of eastern Canada. Right, reflooded cells and breach points, modified from the Canadian High Resolution Digital Elevation Model: http://open.canada.ca/data/en/dataset/957782bf-847c-4644-a757-e383c0057995—licensed under the Open Government Licence Canada: https://open.canada.ca/en/open-government-licence-canada.

The restoration site consists of two marsh cells separated by a tidal gate (Fig 1). The northern cell (A) is ~200 m x 800 m while the southern cell (B) is ~150 m x 650 m. Salt marsh cordgrass (*S*. *alterniflora*) was observed for the first time in both cells in 2012 followed by patch growth via rhizomes after that [32]. At the time of sampling in August 2016, although *S*. *alterniflora* had become established across the restored marsh, areas of exposed mud with no vegetation were still present. A large channel divides cell B into two approximately equal halves; most of the northern half is un-vegetated while dense *S*. *alterniflora* (with cover ranging from 65 to 99%) occupies most of the southern half of the cell. Although patches of bare mud remain in cell A, most of the cell is also covered by dense *S*. *alterniflora* (with cover ranging from 65 to 99%).

The study site belongs to the government of New Brunswick. The government issued permission to work on the site in 2010. There are no known rare or endangered species on the study site.

### Field sampling

**Fig 2:**
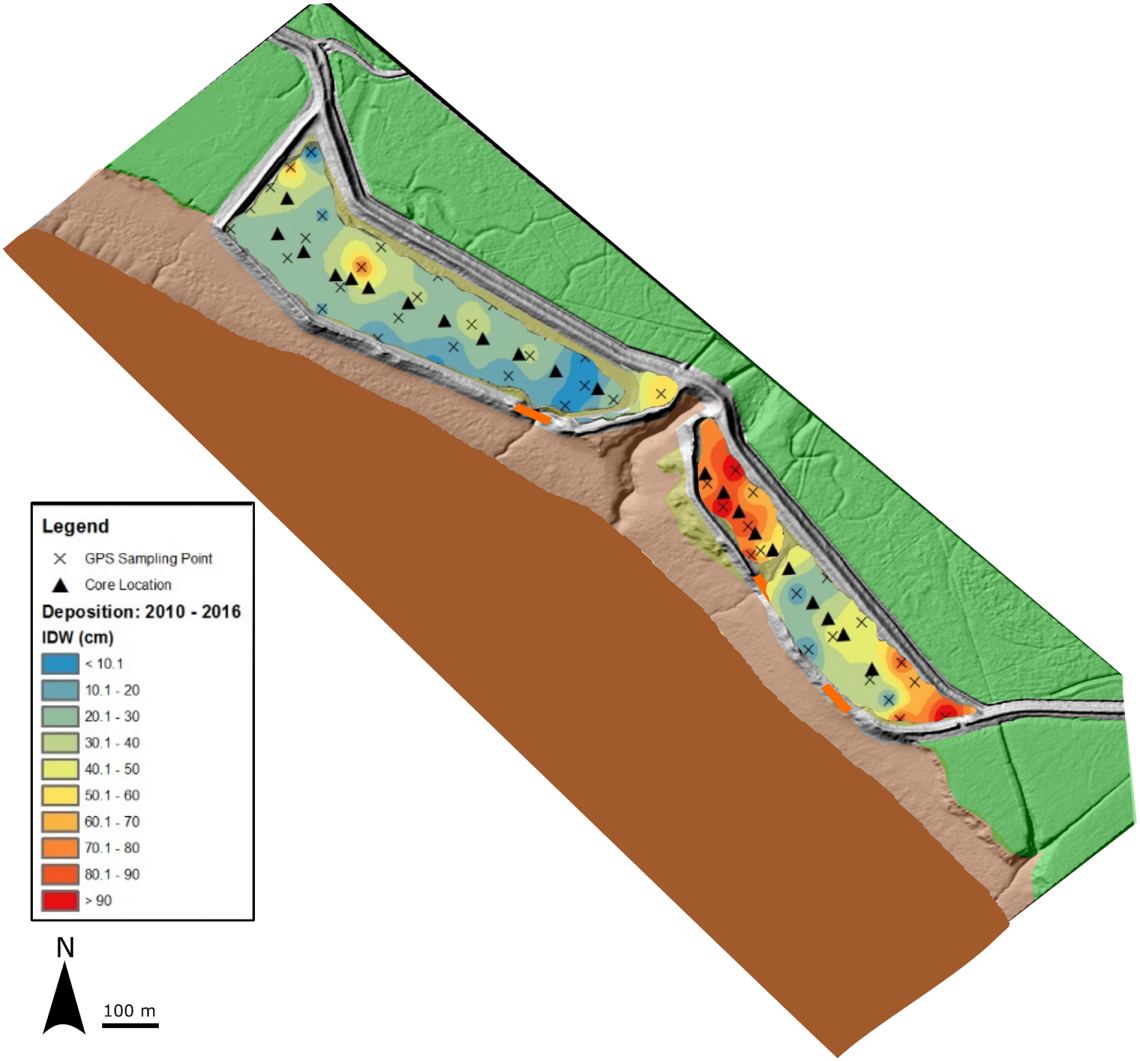
Sample locations and interpolated depth of new deposition from 2010 to 2016. (Base imagery modified from the Canadian High Resolution Digital Elevation Model: http://open.canada.ca/data/en/dataset/957782bf-847c-4644-a757-e383c0057995—licensed under the Open Government Licence Canada: https://open.canada.ca/en/open-government-licence-canada).

The elevation of the ground surface was measured in each cell prior to the breaching of the agricultural dykes in 2010. Elevations were measured yearly in October after breaching at 50 different locations (Fig 2) using a high precision Leica 1200 GPS (±10 mm) with the rover carried by foot at low tide. Deposition was estimated based on yearly differences in the elevation of the ground surface at each location.

Two corers were used to collect soil samples in August 2016, a 25-mm diameter Dutch gauge corer and a Russian peat auger. Both corers are designed to ensure sediment is retrieved with minimal compaction. Soil blocks were also collected using a knife and trowel in sampling locations where it was not possible to recover a sufficient quantity of material using either corer.

Cores were collected along a transect running roughly parallel to the dyke near the center of the cell (Fig 2). Ten cores were collected for loss on ignition (LOI) analysis from each cell. All cores were driven to refusal or until the former agricultural soil layer was reached. At each core site % plant cover was recorded; sampling locations were classified as vegetated if *S*. *alterniflora* cover exceeded 65% within a 3 m radius of core placement. Cores from cell A were collected using the Dutch auger. The unconsolidated nature of the sediment in many areas of cell B required the use of the Russian peat auger.

Newly accumulated sediment was easily distinguished from the former agricultural layer by soil colour. New sediment was light brown (Munsell colour 10YR 4/3 to 10YR 5/1) while the former agricultural layer was grey (Munsell colour 5B 7/1). The new sediment was dense and composed primarily of silt with the occasional presence of organic debris. In many cores a thin (1–3 cm) black layer of plant material was observed immediately below the newly deposited sediment, representing the remains of former pasture vegetation. New sediment deposited above the old agricultural layer was placed in plastic bags for transport to the laboratory. Sediments were stored under refrigeration.

### Lab analyses

Organic matter content of sediments was determined by LOI following the method of Heiri et al. [34]. All samples were oven dried at 60°C until there was no additional mass lost. Sediments were then ground using a mortar and pestle. Sub-samples of approximately 2.5 g were placed in crucibles and dried at 60°C until all moisture was lost and then weighed in preparation for LOI. In a programmable muffle furnace the sub-samples were heated to 350°C for 1 hour followed by 4 hours at 550°C [34]. Samples were removed from the muffle furnace and placed in a desiccator to cool to room temperature prior to weighing. If the difference in mass loss between two replicate samples exceeded 10%, additional replicates were run. To convert from organic matter to organic carbon content we used the conversion factor developed by Craft et al. [35] for salt marshes: Organic C fraction = 0.40(LOI fraction) + (0.025*LOI fraction)^2^.

### GIS analysis and carbon stock calculation

Measurements of sediment depth [S1 Table] were input into ESRI ArcGIS v10.2.2. Both inverse distance weighting (IDW) and Kriging [36] were used to estimate the depth of the new sediment deposited across the marsh. The estimated volume of total sediment deposition from 2010 to 2016 differed by <2% between the two techniques and IDW was thus employed to estimate yearly accumulation rates. Interpolations were constrained to areas that would be flooded by tidal waters between the old and new dykes. Active channels around the perimeter of the developing marsh were excluded because the consistent flow of water into and out of the cells prevented much deposition from occurring. The interpolated surface was thus constrained by establishing polygon boundaries (with a combined area of 309 600 m^2^) based on field observations and expert judgement. ArcGIS’s surface volume tool was used to calculate the total volume of new sediment based on the interpolated depths and the polygon surface areas. Total C stock was calculated by multiplying the mean C density of the sediment (across all the cores collected) by the total volume.

### Statistical analyses

All statistical analyses were performed using IBM SPSS Statistics v22. Independent t-tests were used to assess differences in sediment C density between vegetated and non-vegetated sediment and between cells A and B. The Anderson-Darling method revealed that data did not vary significantly from a normal distribution. Linear regression was used to test the relationship between C storage and depth for each core. Regression was forced through the origin (no intercept) as it is not possible to have negative C storage (nor can organic C be present if there is no sediment).

## Results

**Fig 3:**
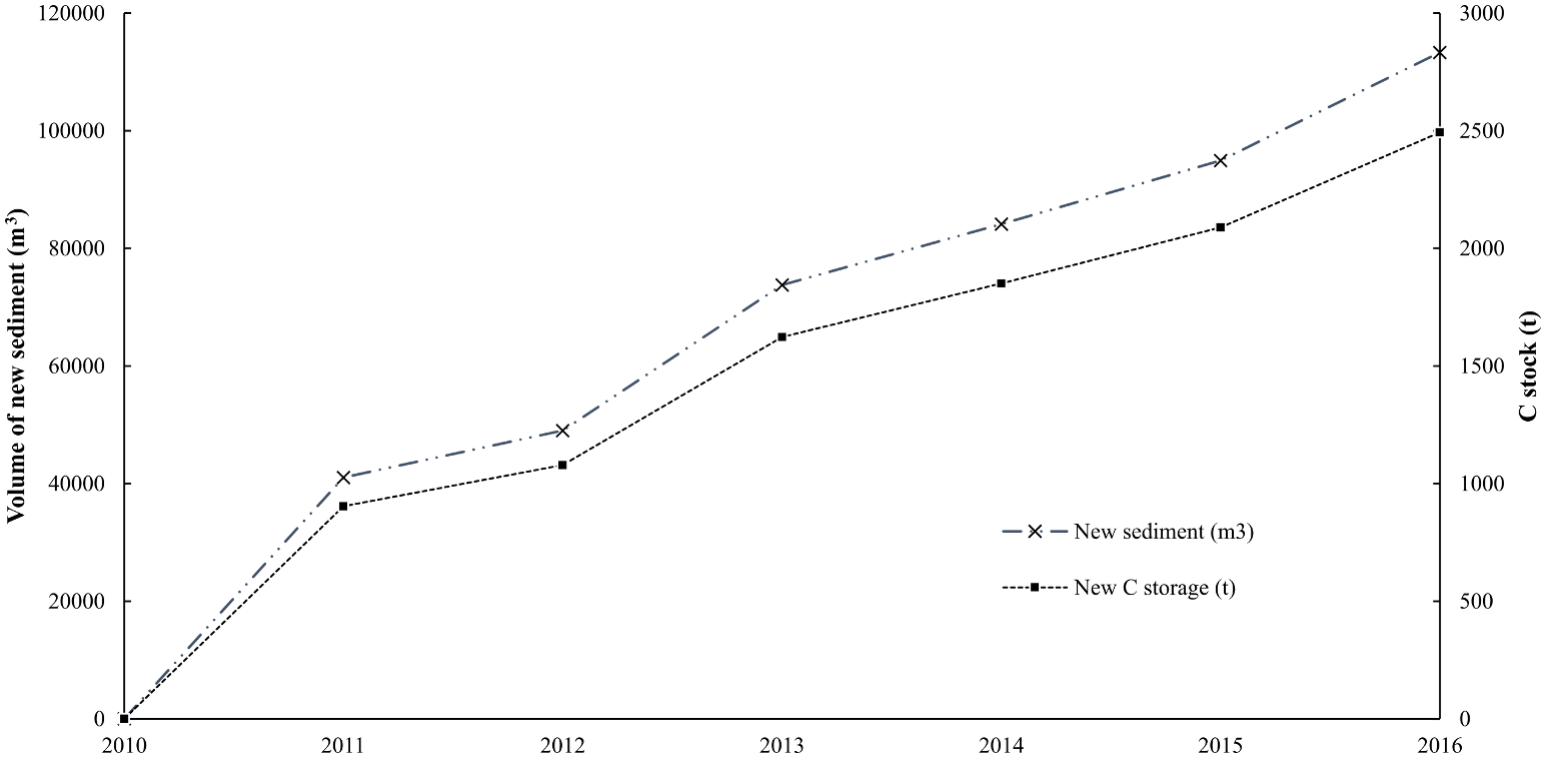
Cumulative sediment deposition and C storage from 2010 to 2016.

**Fig 4:**
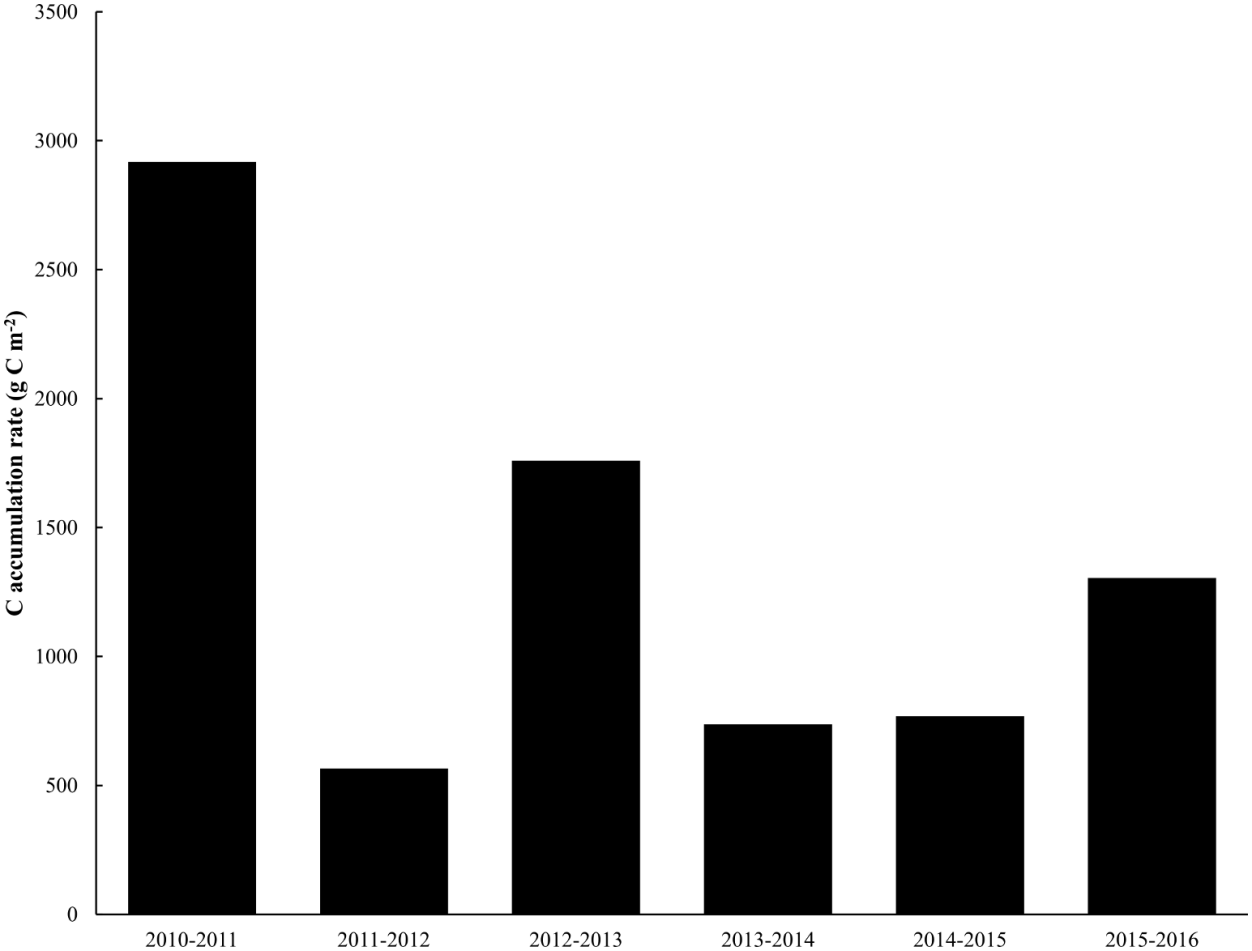
Mean annual C accumulation rates (2010 to 2016) in a recovering marsh.

**Fig 5:**
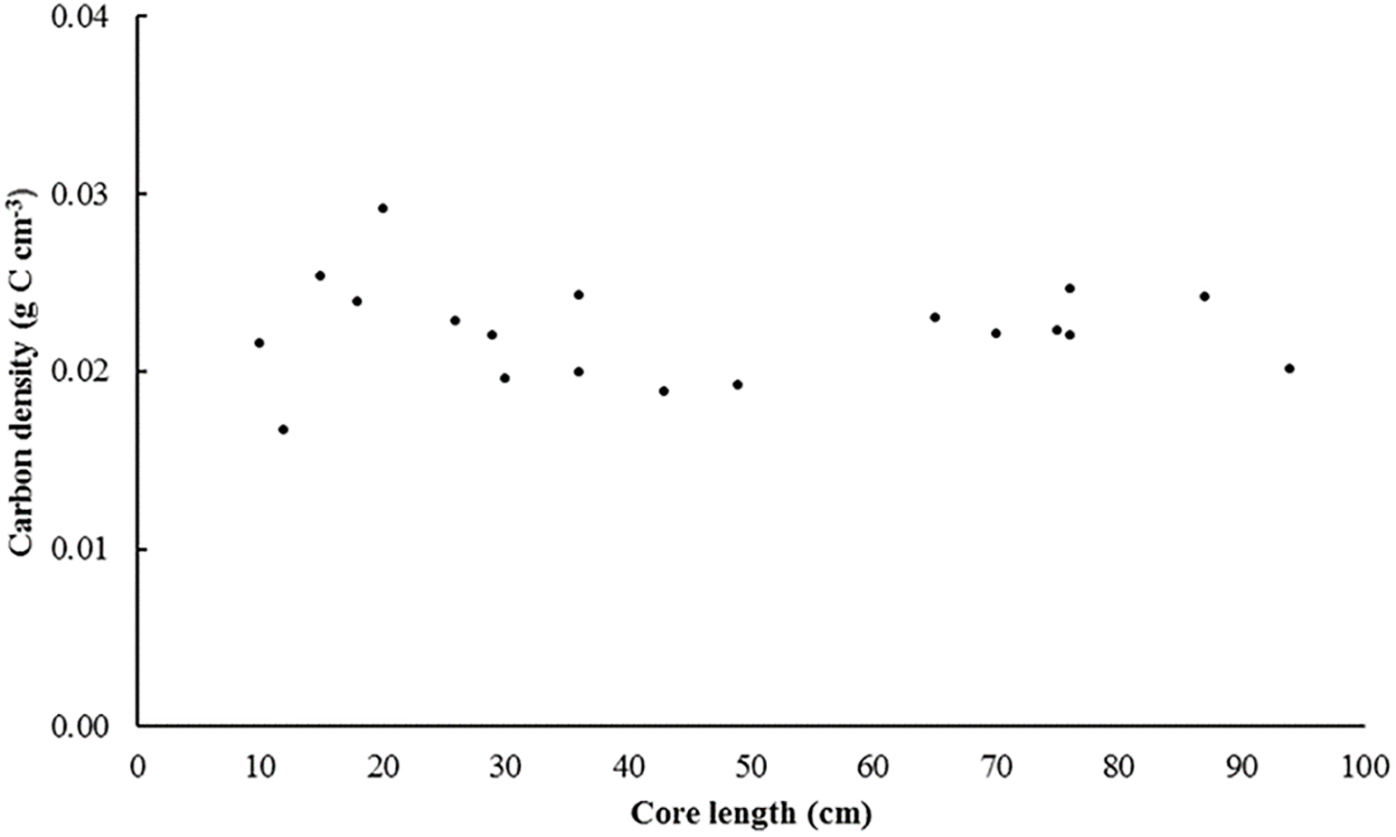
Mean C density vs. total core length in a recovering marsh.

Yearly measurements of elevation of the sediment surface were used to determine depth of new sediment deposition. Sedimentation was rapid following MR with nearly 37% of the 6-year cumulative deposition occurring within the first year. Over the 6 years, depths of new sediment ranged from 3 to 102 cm (Figs 3 and 4). Bulk density and % organic C varied from 0.74 to 1.23 g cm^-3^ and 2.0 to 3.0%, respectively, yielding C densities from 0.019 to 0.029 g C cm^-3^ (Table 1 and S2 Table). The total C stock in the recovering marsh was estimated at 2 493 t C (~9 141 t CO_2_ eq.) with a mean areal storage of 8 052 g C m^-2^. An independent t-test showed mean C density did not differ significantly between marsh cells A (n = 10) and B (n = 10) (p = 0.250). The mean C density measured in each core was relatively constant across cores regardless of core length (Fig 5); Pearson’s correlation test revealed no significant relationship between C density and core length (p = 0.971).

**Table 1:** Average soil properties from cores collected in a recovering marsh.

	Depth range		Bulk density	Organic Carbon	
Core	(cm)		(g cm^-3^)	(%)	g cm^-3^
**A2**	0	26	1.039	2.19	0.023
**A3**	0	30	0.964	2.03	0.020
**A4**	0	43	0.893	2.11	0.019
**A5**	0	70	0.880	2.47	0.022
**A6**	0	20	1.019	2.87	0.029
**A7**	0	29	0.927	2.37	0.022
**A9**	0	75	0.992	2.25	0.022
**A10**	0	10	0.778	2.76	0.022
**A11**	0	15	0.990	2.56	0.025
**A13**	0	18	1.227	1.94	0.024
*B1	41	96	0.874	2.23	0.020
**B2**	0	94	0.902	2.20	0.020
**B3**	0	87	1.055	2.29	0.024
**B4**	0	76	1.141	2.16	0.025
**B5**	0	76	1.072	2.05	0.022
**B6**	0	12	0.735	2.27	0.017
**B7**	0	36	0.965	2.06	0.020
**B8**	0	36	1.105	2.19	0.024
**B9**	0	49	0.928	2.07	0.019
**B10**	0	65	1.046	2.19	0.0230
**Mean**			0.977	2.26	0.022
**Std.dev**			0.116	0.23	0.0027

*It was not possible to retrieve a surface sample from 0–41 cm as the material was too wet to collect a core

No significant differences in C densities were observed between cores collected in bare (n = 6) vs. vegetated (n = 5) areas (p = 0.432). To further investigate the effect of differences in C density within and below the live rooting zone (0–30 cm), samples were also binned into two range classes for analysis: 0-30 cm (n = 8) and >30 cm (n = 6). An independent t-test found no significant difference in C density between samples collected from 0–30 cm and those from depths >30 cm (p = 0.237).

**Fig 6:**
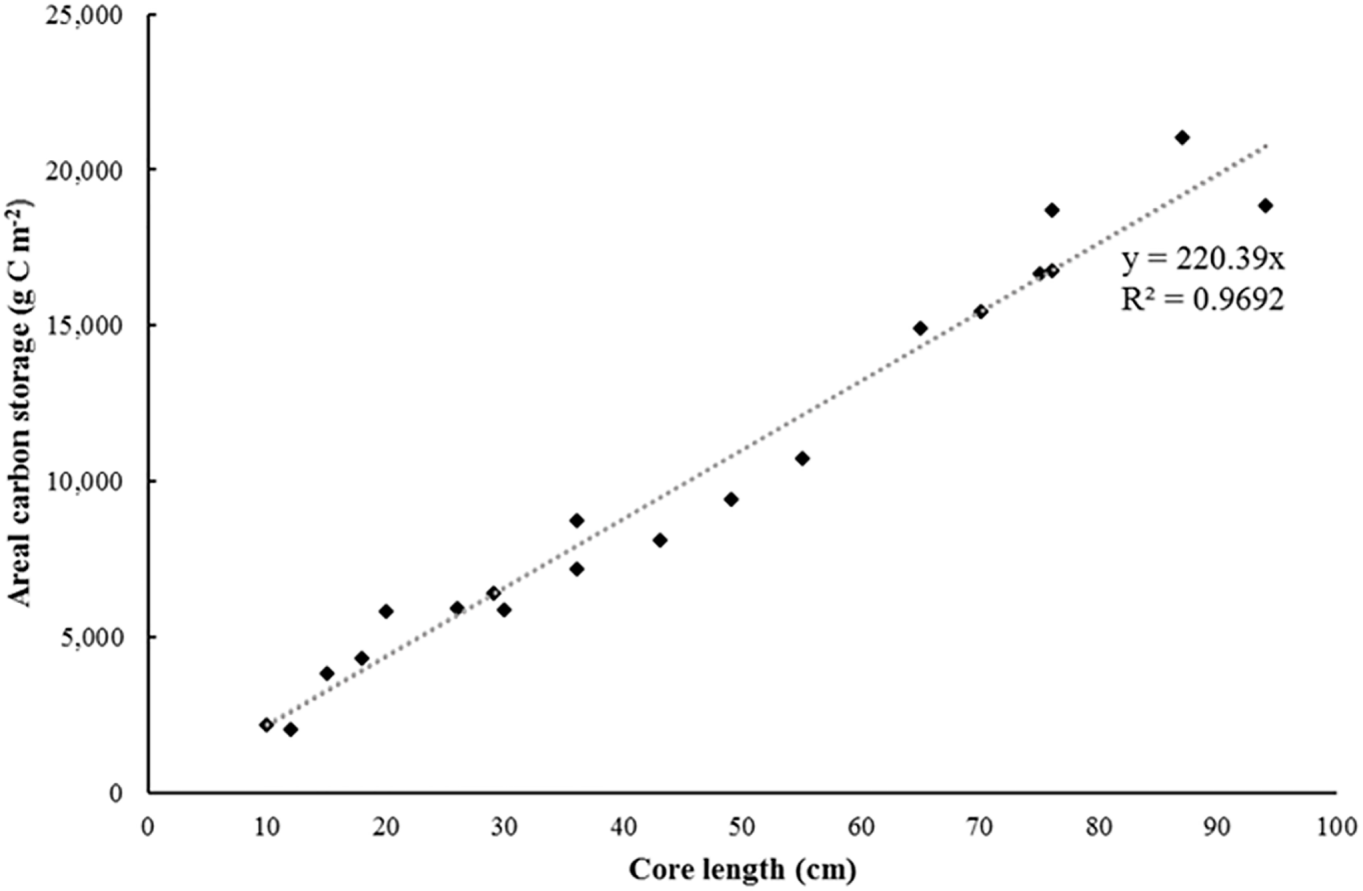
Areal C storage vs. core length in a recovering salt marsh.

Although total C stock was calculated using the mean C density from all cores, areal C storage was also calculated individually for each core based on the mean C density and depth of each core (Fig 6). As expected, due to the relatively consistent C densities measured across the marsh, sediment depth was a significant predictor of areal C storage and explained 96.9% of the variation in new C storage per m^2^ (p<0.001), albeit significance of the relationship is enhanced as sediment depth is a component of the equation used to calculate C storage. The regression analysis yields the equation Y = 220.39*depth(cm) which predicts areal C storage (g C m^-2^) based on the depth of newly accumulated sediment.

## Discussion

The data indicate that C accumulation in the six years following MR was driven primarily by sediment deposition. We assume that the bulk of C in the new marsh deposit is associated with the suspended sediment in the tidal floodwater. Sediment cores collected from the Aulac marsh have relatively uniform C densities which do not vary with depth. These findings are consistent with observations from other Bay of Fundy recovered marshes that show no systematic decrease in soil C density with depth [31]. The mean C density of 0.022 g C cm^-3^ measured in this study is comparable to the mean C density of 0.026 g C cm^-3^ reported for Wood Point marsh located 7 km south of the Aulac site on the same arm of Chignecto Bay [1]. Despite similar soil C densities, the six-year mean C accumulation rate of 1 329 g C m^-2^ yr^-1^ measured at the Aulac MR site is more than 5 times higher than the burial rate of 259 g C m^-2^ yr^-1^ Chmura et al. [1] found accumulated at Wood Point over a 5-year period, and much higher than the rate of 92 g C m^-2^ yr^-1^ reported by Burden et al. [37] at the Tollesbury managed realignment site on Britain’s Blackwater Estuary.

We note that the high sediment and C accumulation rates observed at Aulac within the first 6 years following MR are likely to decrease over time as sediment fills the remaining accommodation space and the marsh accretes. Sediment deposition and C burial rates at Aulac will thus likely converge towards rates similar to other mature marshes in the area.

The suspended sediment load in the Bay of Fundy is composed of sand, silt, clay, plankton, and organic debris, with silt and organic debris being the major components [38]. The mean organic C content of newly deposited sediment measured in this study was 2.3%, within the range of 0.3% to 2.7% reported by Miller [39] for the suspended sediment load in the Bay of Fundy. These findings suggest that in the short term, soil C density is driven primarily by the inherent C content of the incoming sediment. We note, however, that the upper Bay of Fundy is a relatively unique environment due to its exceptionally large tidal amplitude and suspended sediment concentrations. Thus, the results of this study may not be applicable to marshes with lower tidal amplitudes and suspended sediments loads. To develop more broadly applicable models, it will be necessary to understand the role of sediment deposition and allochthonous C. Eventually, the organic C content of local suspended sediment could be used with deposition models to predict C accumulation following restoration. Over time, predictions could be validated by measuring elevation change of a recovering marsh surface using remote sensing techniques that provide digital elevation models, such as Structure from Motion [40]. These methods could reduce monitoring and validation costs and increase the financial value of blue C restoration initiatives.

The practice of preserving the old dykes is likely an important factor in the rapid accumulation of sediment, essentially providing a settling basin for sediments and sheltering them from wave erosion. Although *S*. *alterniflora* was observed as early as 2012, by 2016 areas without vegetation remained. The absence of any significant difference in C density between soil cores collected from bare vs. densely vegetated areas suggests that marsh macrophytes are not major direct contributors to soil organic C in the early stages of recovery following MR. The absence of any relationship between C density and core length further supports this finding; if *S*. *alterniflora* were contributing significantly to the new C storage, deeper sediment deposited before salt marsh plants became established should have a lower C density. However, the vegetation that is present contributes to C storage indirectly as marsh vegetation makes sediment trapping even more effective [41].

The bulk of the C buried in the recovering marsh at Aulac is assumed to be allochthonous, originating from suspended sediment in the Bay rather than from macrophyte growth in the marsh. To demonstrate the net global cooling potential (GCP) of a marsh restoration project, however, it must be established that, in the absence of the project, either the C contained in the suspended sediment load would have been remineralized and returned to the atmosphere as CO_2_ or that C degradation in the restored marsh is slower than in the system in which it would otherwise have been stored [42]. For example, Berhe et al. [43] demonstrated that the process of soil erosion can provide a C sink when eroded material containing C is transported to a new depositional environment with lower rates of decomposition than that from which it was eroded.

If not deposited in marshes, the organic C in the suspended sediments in the upper Bay of Fundy is likely to be deposited in nearby mudflats. Unlike salt marshes, which tend to be stable or accreting, mudflats are highly dynamic systems subject to frequent erosion events, with scouring to depths of 20 cm or more [44]. In the Bay of Fundy, mudflats are subject to intense wave activity and can be incised by deep meandering tidal channels [38]. Bay of Fundy waters are well mixed [38] which enhances mineralization of organic C via increased oxygen delivery to microorganisms [45]. Sediment and associated organic C is also more likely to be preserved in marshes compared with mudflats due to the stabilization effect of macrophyte roots and the associated erosion protection.

A portion of the suspended sediment load in the Bay of Fundy is transported out of the Bay and into the deeper waters of the Gulf of Maine [38]. Del Giorgio and Duarte [46] note that excess organic C which is not stored in coastal environments and exported to the open ocean is oxidized by microorganisms. Duarte et al. [47] suggest that nearly all the organic C received from the coastal environment is respired in the open ocean rather than buried there. Restoring marshes in the Bay of Fundy can regenerate their capacity for C burial and prevent the potential export of suspended sediment and associated C for conversion to CO_2_ in the open ocean, thus satisfying the requirements of the Verified Carbon Standard.

Our research has identified variables that can be used in relatively simple models to simulate short-term C accumulation in flooded dykelands which could be applied to dykeland sites assessed as suitable for restoration by digital terrain and GIS modeling e.g. [48, 49]. The organic C content of local suspended sediment could be used in deposition models to predict C accumulation following MR. Validation of these predictions after restoration will improve future models. These methods could reduce monitoring and validation costs and increase the financial value of blue C restoration initiatives.

### Quantifying the financial value of new carbon storage

The financial value of a restoration project can vary depending on the applicable C price. Within just six years the Aulac MR project accumulated 2 493 t C (~9 141 t CO_2_ eq.). Based on California’s 2017 C price, the MR project could thus have generated ~US$124 000 worth of offset credits in California’s regulated market vs. only ~US$30 000 in the voluntary market. Tidal wetland restoration projects have not yet been recognized as an approved offset methodology in any regulated C market; incorporating tidal wetlands into regulated C markets could greatly increase funding to support restoration activities. When all ecosystem services are considered, restoring degraded marshes may provide value-added benefits well in excess of restoration costs [3].

At present, there are an estimated 330 km^2^ of undeveloped dykelands on the Bay of Fundy [18]. The primary land uses in the area consist of agriculture behind dykes, undeveloped forested upland, and low-density residential development. Although it is estimated that 17 km^2^ of dykeland are highly developed [18], much of this drained land remains underutilized and nearly 87 km^2^ of agricultural dykeland is not currently being farmed in New Brunswick and Nova Scotia [50]. In addition, more than half of public dykes in Nova Scotia are within 0.5 m of critical elevations established in the 1960s and all are will be threatened by rising sea level [51]. It is thus increasingly important to examine the long-term viability of maintaining dykes and coastal defences for areas with low-value land uses. Managed realignment offers a cost-effective and prudent management option given the large area of underutilized dykeland and the growing urgency to prepare for sea-level rise [51, 52].

The value in terms of ecosystem services returned and new C stored per dollar spent on MR is likely to be higher in Maritime Canada than in other jurisdictions. Three of the primary costs associated with MR projects are pre-project planning and design, land acquisition costs, and capital costs for breaching existing defences and realigning new ones [53]. Land acquisition represents one of the largest project costs and is relatively inexpensive in the Bay of Fundy compared with Europe. A 2009 report published by the UK Environment Agency, for example, estimated the cost of land acquisition at approximately £12 000 (US$15 700) per hectare for vacant land suitable for MR [53]. In contrast, the assessed value of vacant dykelands located in in Westcock, NB (only 10 km from our study site in Aulac) is below $CAD1 000 (US$780) per hectare [54], an order of magnitude lower than the average value in the UK. Similarly, restored dykelands in the Bay of Fundy are likely to have fewer adjacent properties, or if so, properties with lower value to protect. Stricter regulations and pre-project planning requirements in the UK are also costly. A 2006 study by the Defra Environment Agency, for example, estimated pre-planning costs of £130 000 (US$170 000) for a typical MR project in the UK [55]. Regulators in the UK often require detailed environmental impact assessments, statutory designations, and applications for project permits [53]. Fewer regulations in Maritime Canada and the provinces bordering the Bay of Fundy mean projects can be planned and executed relatively quickly, while cheaper land also means that they can be executed at a lower cost.

## Conclusions

Better knowledge of the short-term C accumulation rates for recovering marshes is essential to strengthen the case for placing future restoration work on the C market. The results of this study indicate that sediment accretion and C storage in the Bay of Fundy occur very rapidly following MR and can greatly exceed C burial rates in mature marshes. Within just six years, the small MR project in Aulac stored a quantity of CO_2_ equivalent to the annual pollution generated by over 2000 passenger vehicles (based on an emission factor of 4.42 t CO_2_ yr^-1^ for a passenger vehicle [56]). Short term C accumulation in the recovering marsh appears to be driven primarily by the rate of sediment delivery and the C content of the suspended sediment being deposited. Carbon density varied little with depth resulting in a strong linear relationship between sediment depth and C storage; rates of C accumulation in similar macro-tidal systems could thus be estimated using only sediment accretion rates and the mean organic C content of the incoming suspended sediment. Going forward, this could encourage planners to include the financial and climate benefits of blue carbon when assessing MR projects in the Bay of Fundy.

## Supporting information

S1 Table GPS elevations. (XLSX)

S2 Table Measurements of carbon density. (XLSX)
